# Efficacy of Janus Kinase Inhibitors in Alopecia in Jordanian Patients: A Retrospective Cohort Study

**DOI:** 10.7759/cureus.84321

**Published:** 2025-05-18

**Authors:** Asem Aldebei, Yara S Hammouri, Salah A Abdallat, Anees Hjazeen, Alsharif M Muhanna, Hadeel Arnous, Rama Alshwayyat, Mohammad Alkotob

**Affiliations:** 1 Dermatology, Royal Medical Services, Amman, JOR; 2 Community Health Nursing and Biostatistics, Royal Medical Services, Amman, JOR; 3 Dermatology, Private Practice, Amman, JOR; 4 Medicine and Surgery, Al-Balqa Applied University, Amman, JOR

**Keywords:** alopecia areata, baricitini, janus kinase inhibitors, salt score, tofacitinibb

## Abstract

Introduction

Alopecia areata (AA) is an autoimmune disorder causing non-scarring hair loss on the scalp, eyebrows, eyelashes, and other areas. Janus kinase (JAK) inhibitors have emerged as promising treatments, but data on their efficacy in Middle Eastern populations, including Jordanians, are limited. The Severity of Alopecia Tool (SALT) score is commonly used to assess disease severity, while Clinician-Reported Outcome (ClinRO) measures provide additional insights.

Aim

To evaluate the efficacy of JAK inhibitors in Jordanian AA patients using the SALT score as the primary outcome measure.

Methods

A retrospective cohort study was conducted at King Hussein Hospital, Jordanian Royal Medical Services, from January 2020 to December 2023. Medical records of AA patients aged ≥18 years treated with JAK inhibitors were reviewed. Data included demographics, disease duration, previous treatments, and adverse effects. Efficacy was assessed by the percentage change in SALT scores at six and 12 months. Statistical analyses included repeated-measures MANCOVA (Multivariate Analysis of Covariance), Chi-square, and independent t-test. A p-value <0.05 was considered significant.

Results

Our analysis included 57 patients, of which 31 (54.4%) received tofacitinib and 26 (45.6%) received baricitinib. A significantly higher proportion of baricitinib users had treatment durations >12 months (53.8%) compared to tofacitinib users (12.9%), while shorter durations (three to six months) were more common among tofacitinib users (41.9% vs. 15.4%; *p* = 0.003). Baricitinib users showed greater improvement in SALT scores between six to 12 months (92.77% vs. 82.93%; *p* = 0.030, partial η² = 0.084), with a trend toward greater total improvement at 12 months (96.64% vs. 93.11%; *p* = 0.055, partial η² = 0.067). Although not statistically significant, baricitinib showed numerically higher ClinRO improvement in eyebrows from six to 12 months (84.58% vs. 70.29%; *p* = 0.212) and in eyelashes (83.92% vs. 73.40%; *p* = 0.313), suggesting better late-stage response compared to tofacitinib.

Conclusion

JAK inhibitors demonstrated efficacy in Jordanian patients with alopecia areata, leading to enhanced SALT scores and noticeable hair regrowth, with baricitinib demonstrating greater improvement in SALT scores compared to tofacitinib.

## Introduction

Alopecia areata (AA) is an autoimmune condition characterized by non-scarring hair loss which can affect patients psychologically and quality of life [1]. AA affects approximately 0.1%-0.2% of the worldwide population [2]. It is associated with several conditions including atopic dermatitis, vitiligo, and systemic lupus erythematosus [3]. Besides autoimmune factors, AA has genetic components. Studies show that the condition runs in families with an incidence of 7%-18% depending on the type of AA [4]. This disorder happens due to the immune-mediated destruction of hair follicles, involving CD8+ T cells and inflammatory cytokines such as interferon-gamma and interleukin 15 [5]. AA was found to be associated with several human leukocyte antigens, including DQ3, DR4, DR11, and DQ7 [6]. Some treatments like corticosteroids and immunotherapy are usually used in this condition, but some studies have shown that these treatments lack long-term efficacy [7]. Recent advancements in the treatment of alopecia areata showed the emergence of Janus kinase (JAK) inhibitors as a promising treatment option [8]. JAK inhibitors, such as tofacitinib, ruxolitinib, and baricitinib, interfere with the JAK-STAT (JAK-signal transducers and activators of transcription) signaling pathway, which is crucial in the pathogenesis of AA [9].

Patients who receive JAK inhibitors experience hair regrowth with improvements assessed using the Severity of Alopecia Tool (SALT) score and the Clinician-Reported Outcome (ClinRO) measure for eyebrow and eyelash regrowth [8]. However, limited data are available on their efficacy among the Middle Eastern population including Jordanians. Different patient populations can affect the efficacy of JAK inhibitors, due to genetic and environmental factors influencing treatment response [10]. Investigating AA patients’ responses to JAK inhibitors will help improve treatment options for the Jordanian population.

This study aimed to evaluate the clinical response of Jordanian patients with alopecia areata to JAK inhibitors, specifically tofacitinib and baricitinib, by measuring improvements in the SALT score and ClinRO assessments of eyebrow and eyelash regrowth. We hypothesize that baricitinib is associated with greater late-stage improvement compared to tofacitinib. By addressing the current gap in regional data, this study aims to provide evidence-based insights that may inform treatment decisions for AA patients in the Middle East.

## Materials and methods

Study design

This study design was a retrospective observational analysis conducted on patients aged 18 years or older. The study aimed to evaluate the efficacy of JAK inhibitors in Jordanian patients with alopecia areata using the SALT score as a primary outcome measure. Data were collected from the medical records of Jordanian AA patients treated with JAK inhibitors.

Data collection

A total of 57 patients aged 18 years or older. Patient demographics, treatment details, and clinical outcomes were extracted from medical records and recorded in a structured database for statistical analysis. The collected variables included: age, gender, treatment duration, dosage, SALT score, and ClinRO measures. This study included patients aged 18 years or older with a confirmed diagnosis of alopecia areata who were treated with JAK inhibitors and had complete clinical records, including SALT scores. Patients were excluded if they had missing clinical data, incomplete follow-up information, or if they were prescribed JAK inhibitors for conditions other than alopecia areata.

Ethical consideration

This study approval was obtained by the Institutional Review Board (IRB) committee in King Hussein Hospital, Jordanian Royal Medical Services (approval no:2/2025). This study was conducted in accordance with the Declaration of Helsinki (1964). Approval was obtained before data collection.

Statistical analysis

The categorical data were represented using frequencies and percentages, whereas the scale data was expressed using mean and standard deviation. The Chi-square test or Fisher-Freeman-Halton Exact Test, as appropriate, was used to explore the distribution of patients’ gender, doses, and duration of treatment between JAK inhibitor groups. An independent t-test was utilized to assess mean differences in patients’ age between JAK inhibitor groups.

To determine whether the percentage of improvement in SALT score, CLinRO eyebrows score, and CLinRO eyelashes score were statistically significant between JAK inhibitors over time, a general linear model (repeated measures MANCOVA) was utilized, which allows us to investigate changes in percentage scores while controlling covariates. Furthermore, partial Eta-squared was reported as effect size, P-value less than 0.05 was deemed statistically significant. Data analysis was conducted using IBM SPSS Statistics for Windows, Version 28 (IBM Corp., Armonk, NY).

## Results

The patients' demographic and treatment variables were correlated with (JAK) inhibitors. A statistically significant differences were found between tofacitinib and baricitinib users in terms of treatment duration, (P = 0.003). A higher proportion of patients using baricitinib had been on treatment for more than 12 months (53.8%) compared to those using tofacitinib (12.9%). Conversely, a larger percentage of tofacitinib users were on treatment for shorter durations, particularly in the three to six months category (41.9% vs. 15.4%). On the other hand, variables, including gender distribution (P = 0.217) and age (P = 0.292), did not show a statistically significant difference between the two groups. The gender distribution was relatively comparable, with males being slightly more prevalent among baricitinib users (61.5%) than tofacitinib users (45.2%). The mean age was also similar between groups, with no significant difference noted. A comparison of demographic and treatment variables among JAK inhibitor users is shown in Table 1.

**Table 1:** Comparison of demographic and treatment variables among Janus kinase inhibitor users. X^2^: Chi-square, FH: Fisher-Freeman-Halton Exact Test, t: independent t-test, SD: standard deviation.

Variables	Categories	Janus kinase inhibitors		Test value	P-value
		Tofacitinib n=31 (54.4%)	Baricitinib n=26 (45.6%)		
Age/years (Mean±SD)	25.46±9.60	26.73±9.18	24.0±10.04	1.064	0.292 ^t^
Gender n(%)	Females	17 (54.8)	10 (38.5)	1.521	0.217 ^X2^
	Males	14 (45.2)	16 (61.5)		
Duration of treatment n(%)	< 3 months	10 (32.3)	3 (11.5)	13.723	0.003 ^FH^
	3-6 months	13 (41.9)	4 (15.4)		
	6-12 months	4 (12.9)	5 (19.2)		
	>12 months	4 (12.9)	14 (53.8)		

To abstract the percentage of change in SALT scores, the results of the MANCOVA test provided in Table 2 revealed that the overall improvement from baseline to 12 months showed a trend toward significance (P = 0.055, partial eta squared = 0.067). Baricitinib users exhibited slightly greater improvement (96.64%) compared to tofacitinib users (93.11%), with marginal statistical significance (P = 0.055). However, the improvement from baseline to six months did not show a statistically significant difference (P = 0.141, partial eta squared = 0.04). Baricitinib users had a higher mean improvement (67.76%) compared to tofacitinib users (59.33%). Conversely, there is a statistically significant difference in SALT score improvement from six months to 12 months between baricitinib and tofacitinib users (P = 0.030, partial eta squared = 0.084). Baricitinib users showed a greater improvement (92.77%) compared to tofacitinib users (82.93%).

**Table 2:** Comparison of SALT score improvement between Janus kinase inhibitors over time using repeated measures of MANCOVA. SALT: Severity of Alopecia Tool, MANCOVA: Multivariate Analysis of Covariance, SD: standard deviation. * Mean adjusted by covariates.

Percentage of SALT score improvement	Janus kinase inhibitors	*Mean of improvement %	SD	p-value	Partial Eta-squared	95% CI for difference	
Baseline to 6 months	Baricitinib	67.76	18.04	0.141	0.04	-2.87	17.74
	Tofacitinib	59.33	22.70				
6 months to 12 months	Baricitinib	92.77	12.10	0.030	0.084	0.98	18.71
	Tofacitinib	82.93	19.63				
Baseline to 12 months	Baricitinib	96.64	6.26	0.055	0.067	-0.08	7.13
	Tofacitinib	93.11	7.00				

**Figure 1:**
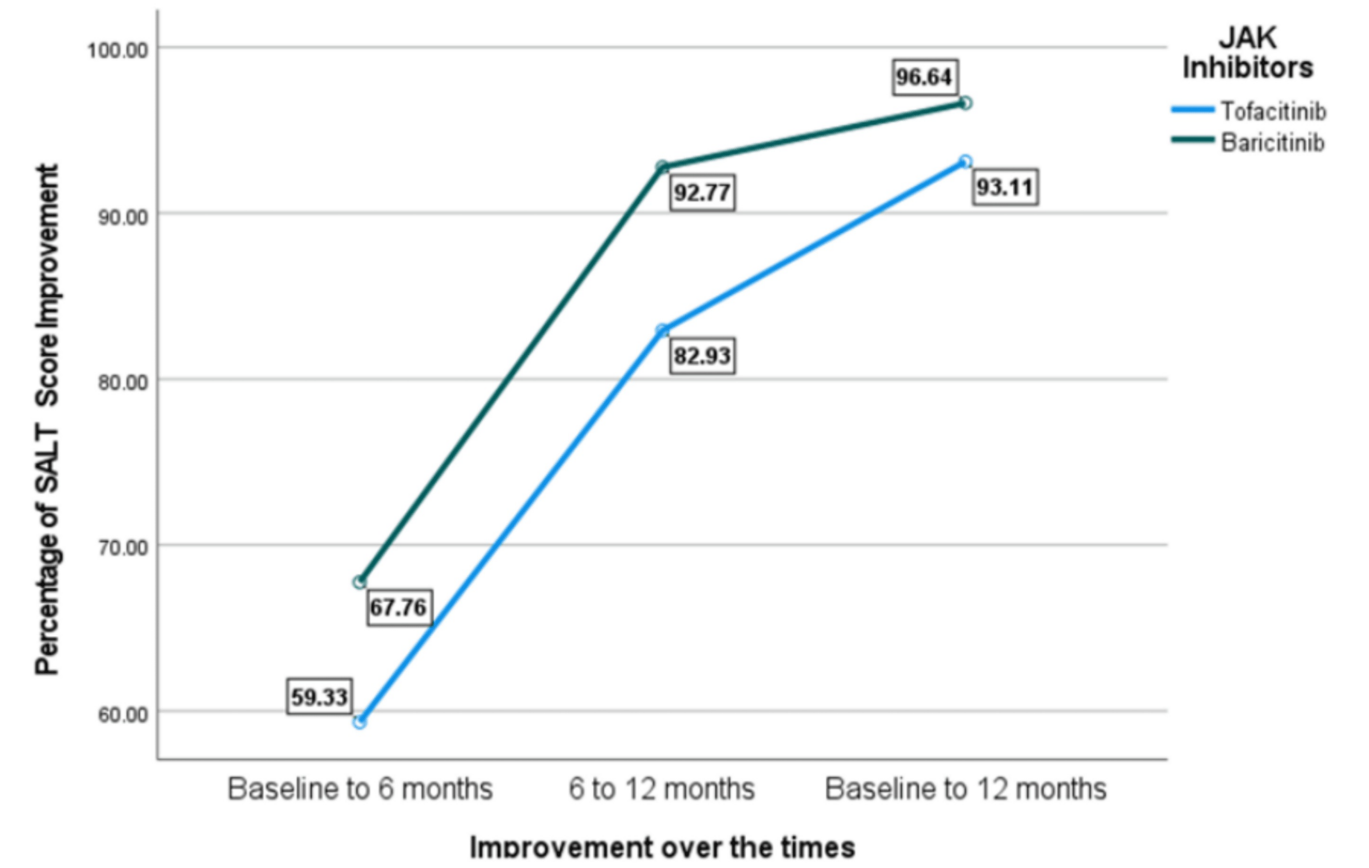
Percentage of SALT score improvement. JAK: Janus kinase, SALT: Severity of Alopecia Tool.

Furthermore, Figure 1 illustrates the percentage improvement in SALT scores over time (from baseline to 12 months) among patients treated with two JAK inhibitors, Both JAK inhibitors led to progressive improvement in SALT scores over 12 months. However, baricitinib consistently demonstrated greater improvement at all time points, reaching 96.64% vs. 93.11% for tofacitinib by month 12, suggesting a more substantial late-stage response of scalp hair regrowth in the baricitinib group.

As regards the ClinRO eyebrows score improvement, the results presented in Table 3 did not show statistically significant differences between baricitinib and tofacitinib users at any time point. However, baricitinib users exhibited numerically higher improvements, particularly in the later stages of treatment. From baseline to six months, tofacitinib users had a slightly greater mean improvement (38.77%) compared to baricitinib users (34.01%, P = 0.541). In contrast, from six to 12 months, baricitinib demonstrated a larger increase (84.58%) compared to tofacitinib (70.29%, p = 0.212). By 12 months, baricitinib users achieved an overall improvement of 90.25%, while tofacitinib users reached 81.52% (P = 0.188).

**Table 3:** Comparison of CLinRO eyebrows score improvement between Janus kinase Inhibitors over time using repeated measures of MANCOVA. ClinRO: Clinician-Reported Outcome, MANCOVA: Multivariate Analysis of Covariance, SD: standard deviation. * Mean adjusted by covariates.

Percentage of CLinRO eyebrows score improvement	Janus kinase inhibitors	*Mean of improvement %	SD	p-value	Partial Eta-squared	95% CI for difference	
Baseline to 6 months	Baricitinib	34.01	23.57	0.541	0.009	-10.82	20.34
	Tofacitinib	38.77	39.33				
6 months to 12 months	Baricitinib	84.58	29.95	0.212	0.036	-8.46	37.04
	Tofacitinib	70.29	35.03				
Baseline to 12 months	Baricitinib	90.25	19.65	0.188	0.040	-4.43	21.89
	Tofacitinib	81.52	18.93				

**Figure 2:**
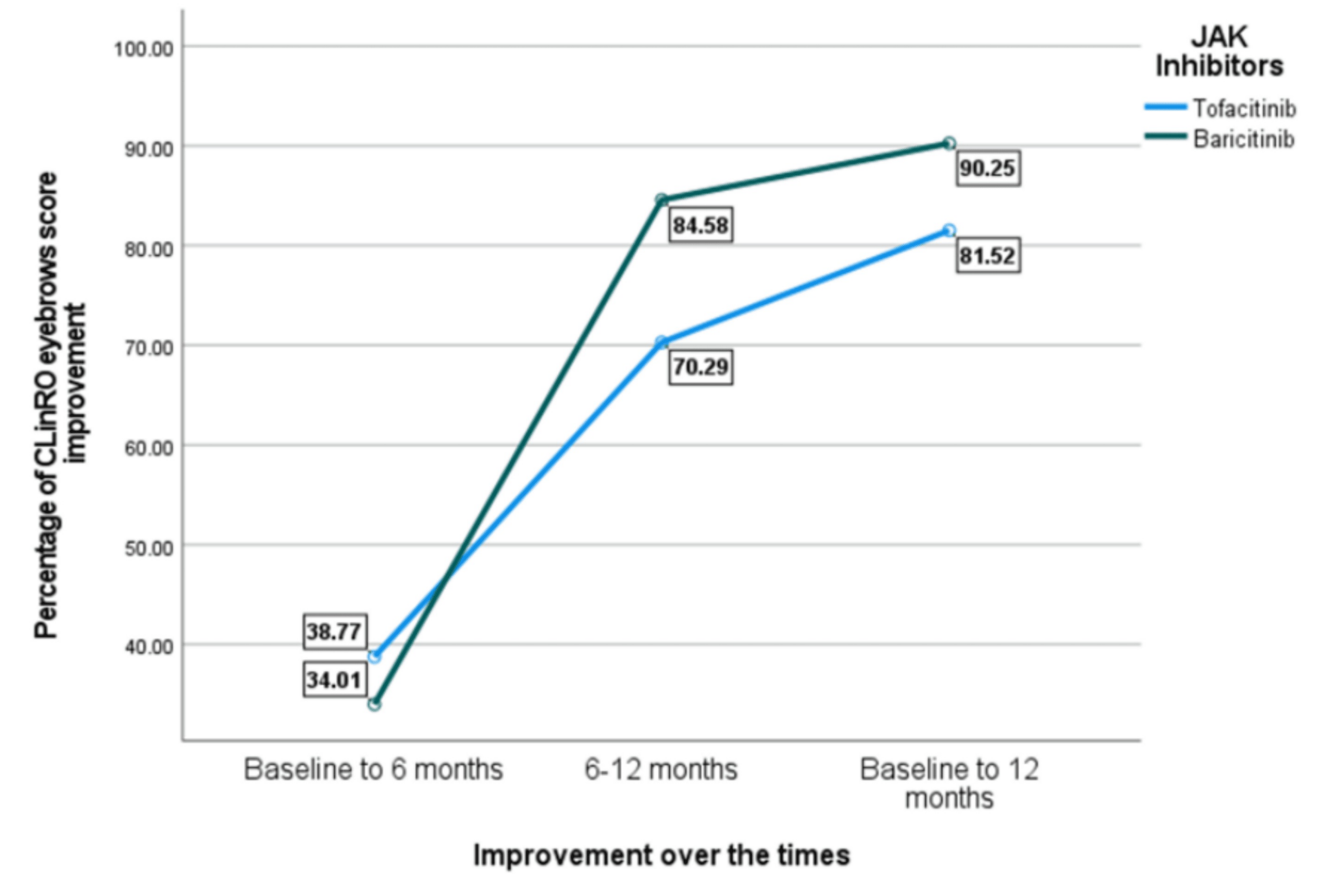
Percentage of CLinRO eyebrows score improvement. JAK: Janus kinase, ClinRO: Clinician-Reported Outcome.

Moreover, Figure 2 demonstrates that baricitinib showed higher improvement in eyebrow regrowth across all time intervals, with the most pronounced difference noted between months 6-12 (84.58% vs. 70.29%). By month 12, baricitinib reached 90.25% improvement, compared to 81.52% with tofacitinib.

The ClinRO eyelash score improvement did not show statistically significant differences between baricitinib and tofacitinib users at any time point (Table 4). From baseline to six months, the mean improvement was similar between the two groups, with tofacitinib users showing (34.81%) improvement and baricitinib users showing 33.31% (P = 0.842). During the six- to 12-month period, baricitinib users had a higher mean improvement (83.92%) compared to tofacitinib users (73.40%), but this difference was not statistically significant (p = 0.313). By 12 months, baricitinib users had a total improvement of 87.62%, while tofacitinib users reached 79.61% (p = 0.346).

**Table 4:** Comparison of CLinRO eyelashes score improvement between Janus Kinase Inhibitors over time using repeated measures of MANCOVA. ClinRO: Clinician-Reported Outcome, MANCOVA: Multivariate Analysis of Covariance, SD: standard deviation. * Mean adjusted by covariates.

Percentage of CLinRO eyelashes score improvement	Janus kinase inhibitors	*Mean of improvement %	SD	p-value	Partial Eta-squared	95% CI for difference	
Baseline to 6 months	Baricitinib	33.31	23.12	0.842	0.001	-13.65	16.66
	Tofacitinib	34.81	22.74				
6 months to 12 months	Baricitinib	83.92	31.06	0.313	0.025	-10.28	31.33
	Tofacitinib	73.40	30.08				
Baseline to 12 months	Baricitinib	87.62	23.49	0.346	0.022	-8.98	25.00
	Tofacitinib	79.61	26.09				

**Figure 3:**
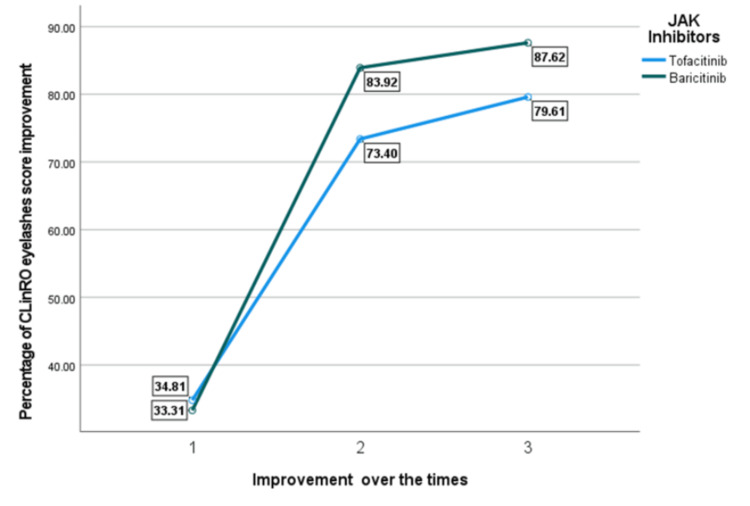
Percentage of CLinRO eyelashes score improvement. JAK: Janus kinase, ClinRO: Clinician-Reported Outcome.

Figure 3 illustrates that the Improvements in eyelash scores were primarily comparable, but baricitinib established a sharper increase after the first time point. By the end of the study, baricitinib attained 87.62% improvement versus 79.61% with tofacitinib.

## Discussion

Alopecia areata (AA) is a chronic autoimmune disorder, presenting as a nonscarring alopecia of the scalp and other areas of the body. It is a common condition, affecting up to 2% of the global population [11]. AA can result in a significant psychological and financial burden on society [12]. A severe form of this condition is believed to be associated with younger onset, along with nail changes, or a family history of autoimmune disorders [13]. The severity of this disease can be evaluated by using clinical tools such as the SALT score. A SALT score equal to or higher than 20 is an indicator of receiving systemic therapy in AA [14]. It was believed that corticosteroids were the preferred first-line treatment for AA followed by immunotherapy [15]. However, recent studies reported that biological therapies, especially JAK inhibitors, show the most promising results for providing effective treatment of AA [16]. Baricitinib's selectivity for JAK1 and JAK2 may contribute to its efficacy. This selectivity allows for more targeted immunomodulation which is significant in autoimmune conditions like AA. However, tofacitinib inhibits JAK1 and JAK3, which might result in a different therapeutic profile [17].

In this study, we aimed to evaluate the efficacy of JAK inhibitors in Jordanian patients with AA using the SALT score as a primary outcome measure. Our results reported that patients treated with baricitinib tended to have longer treatment periods. This aligns with findings from the two phase III trials, where baricitinib's efficacy in severe AA patients was higher over time with 40.9% achieving hair regrowth after 52 weeks of continuous treatment [18]. In our cohort, the mean age of the patients receiving tofacitinib was 25.46 years and for the baricitinib group, it was 26.73 years.

In our study, both JAK inhibitors have led to significant improvements in SALT scores (p = 0.055) over a 12-month period. However, baricitinib showed a greater improvement (96.64%) between six and 12 months compared to tofacitinib (93.11%), with marginal statistical significance (p = 0.055). However, the improvement from baseline to six months did not show a statistically significant difference (p = 0.141). Baricitinib users had a higher mean improvement (67.76%) compared to tofacitinib users (59.33%), but the difference was not statistically significant. A recent study indicated that 32% of patients had a SALT score of 20 or less after six months of receiving baricitinib, whereas only 12% reached this outcome with other therapies [19]. Our cohort reported both JAK inhibitors improved eyebrow regrowth over 12 months. However, baricitinib showed slightly better results. Findings from the BRAVE-AA1 and BRAVE-AA2 phase 3 trials demonstrated that baricitinib improved eyebrow regrowth in patients with severe AA [20]. Moreover, another retrospective study indicated that 60% of patients with alopecia universalis experience eyebrow and eyelash regrowth after switching to baricitinib after inadequate response to tofacitinib [21]. These studies supported the role of baricitinib in improving eyebrow regrowth in AA patients. Our study showed that both baricitinib and tofacitinib improved eyelash growth over 12 months with baricitinib slightly having higher improvement. Results from a randomized controlled study reported that at 36 weeks, more patients treated with baricitinib had eyelash regrowth compared to those on placebo. They significantly improved with 4 mg baricitinib [22].

Implication

To the best of our knowledge, this is the first study to conduct a head-to-head comparison between baricitinib and tofacitinib in a Middle Eastern population. These findings could support treatment decisions in Jordan and the Middle East, where the availability and cost of medications matter. The slight benefit of baricitinib might help doctors choose the most suitable treatment for their patients. This study has some limitations including the retrospective nature of the study and the relatively small sample size which limited the generalizability of the results. Additionally, the follow-up period was limited to 12 months. Future studies should include larger samples and longer follow-up periods to assess the long-term efficacy and safety of JAK inhibitors in AA.

## Conclusions

This study highlights the role of JAK inhibitors in improving hair regrowth in alopecia areata patients. Both baricitinib and tofacitinib showed improvements over time, with baricitinib having slightly better results. Our results indicate that JAK inhibitors are effective in promoting hair regrowth in various areas, such as the scalp, eyebrows, and eyelashes. We also reported variation in response to treatment based on factors such as age, sex, and disease chronicity. More studies using larger sample sizes, and longer follow-up periods are required to fully understand the long-term effects and the best ways to treat them. Generally, JAK inhibitors are a useful treatment choice for alopecia areata, with the potential to significantly increase hair growth.
